# Crystal structure of mevalonate 3,5-bisphosphate decarboxylase reveals insight into the evolution of decarboxylases in the mevalonate metabolic pathways

**DOI:** 10.1016/j.jbc.2022.102111

**Published:** 2022-06-09

**Authors:** Mizuki Aoki, Jeffrey Vinokur, Kento Motoyama, Rino Ishikawa, Michael Collazo, Duilio Cascio, Michael R. Sawaya, Tomokazu Ito, James U. Bowie, Hisashi Hemmi

**Affiliations:** 1 Department of Applied Biosciences, Graduate School of Bioagricultural Sciences, Nagoya University, Furo-cho, Nagoya, Aichi, Japan; 2 Department of Chemistry and Biochemistry, UCLA-DOE Institute, Molecular Biology Institute, University of California Los Angeles (UCLA), Los Angeles, California, USA; 3 Departments of Biological Chemistry, UCLA-DOE Institute of Genomics and Proteomics, University of California Los Angeles (UCLA), Los Angeles, California, USA; 4 UCLA-DOE Institute of Genomics and Proteomics, Howard Hughes Medical Institute, University of California Los Angeles (UCLA), Los Angeles, California, USA

**Keywords:** mevalonate pathway, archaea, isoprenoid, crystal structure, mutagenesis, molecular evolution, ATPγS, adenosine 5ʹ-*O*-(3-thio)triphosphate, DMD, diphosphomevalonate decarboxylase, FPP, farnesyl diphosphate, GGPP, geranylgeranyl diphosphate, IP, isopentenyl phosphate, IPP, isopentenyl diphosphate, M3K, MVA 3-kinase, M3P5K, mevalonate 3-phosphate 5-kinase, MBD, mevalonate 3,5-bisphosphate decarboxylase, MVA, mevalonate, MVA3,5BP, MVA 3,5-bisphosphate, MVA5P, MVA 5-phosphate, MVA5PP, MVA 5-diphosphate, MVK, MVA kinase, Ni-NTA, nickel-nitrilotriacetic acid, OLA, oleic acid, PDB, Protein Data Bank, PMD, phosphomevalonate decarboxylase, PMK, phosphomevalonate kinase, PtoMBD, *Picrophilus torridus* MVA 3,5-bisphosphate decarboxylase, SsoDMD, *Saccharolobus solfataricus* diphosphomevalonate decarboxylase

## Abstract

Mevalonate 3,5-bisphosphate decarboxylase is involved in the recently discovered *Thermoplasma*-type mevalonate pathway. The enzyme catalyzes the elimination of the 3-phosphate group from mevalonate 3,5-bisphosphate as well as concomitant decarboxylation of the substrate. This entire reaction of the enzyme resembles the latter half-reactions of its homologs, diphosphomevalonate decarboxylase and phosphomevalonate decarboxylase, which also catalyze ATP-dependent phosphorylation of the 3-hydroxyl group of their substrates. However, the crystal structure of mevalonate 3,5-bisphosphate decarboxylase and the structural reasons of the difference between reactions catalyzed by the enzyme and its homologs are unknown. In this study, we determined the X-ray crystal structure of mevalonate 3,5-bisphosphate decarboxylase from *Picrophilus torridus*, a thermoacidophilic archaeon of the order Thermoplasmatales. Structural and mutational analysis demonstrated the importance of a conserved aspartate residue for enzyme activity. In addition, although crystallization was performed in the absence of substrate or ligands, residual electron density having the shape of a fatty acid was observed at a position overlapping the ATP-binding site of the homologous enzyme, diphosphomevalonate decarboxylase. This finding is in agreement with the expected evolutionary route from phosphomevalonate decarboxylase (ATP-dependent) to mevalonate 3,5-bisphosphate decarboxylase (ATP-independent) through the loss of kinase activity. We found that the binding of geranylgeranyl diphosphate, an intermediate of the archeal isoprenoid biosynthesis pathway, evoked significant activation of mevalonate 3,5-bisphosphate decarboxylase, and several mutations at the putative geranylgeranyl diphosphate–binding site impaired this activation, suggesting the physiological importance of ligand binding as well as a possible novel regulatory system employed by the *Thermoplasma*-type mevalonate pathway.

Recent studies of the mevalonate (MVA) pathway in microorganisms have revealed unexpected variety in this important pathway that supplies precursors for the biosynthesis of isoprenoids, the largest family of natural compounds comprising more than 80,000 chemicals (1, 2, 3, 4, 5, 6, 7). The eukaryotic MVA pathway (Fig. 1) was the first one to be characterized and is found in eukaryotes, some bacteria (8), and archaea of the order Sulfolobales (9). A novel variant of the eukaryotic MVA pathway was more recently identified in thermoacidophilic archaea of order Thermoplasmatales (1, 6, 7). Here, we call this pathway the *Thermoplasma*-type MVA pathway. These two pathways are identical from the starting metabolite, acetyl-CoA, up until MVA, from which the two pathways diverge. In the *Thermoplasma*-type pathway, MVA is acted upon by MVA 3-kinase (M3K), which phosphorylates MVA to form MVA 3-phosphate (1, 7, 10). Then MVA 3-phosphate 5-kinase (M3P5K) converts MVA 3-phosphate into MVA 3,5-bisphosphate (MVA3,5BP) (7). MVA3,5BP is then acted upon by MVA 3,5-bisphosphate decarboxylase (MBD), which catalyzes the decarboxylation of MVA3,5BP along with the elimination of the 3-phosphate group to form isopentenyl phosphate (IP) (6). IP kinase then phosphorylates IP to give isopentenyl diphosphate (IPP) (11), which is an essential building unit in isoprenoid biosynthesis, along with its isomer dimethylallyl diphosphate). The steps outlined here are distinct from the reactions of the eukaryotic MVA pathway catalyzed by MVA kinase (MVK), phosphomevalonate kinase (PMK), and diphosphomevalonate decarboxylase (DMD) outlined in Figure 1 (8).

**Figure 1:**
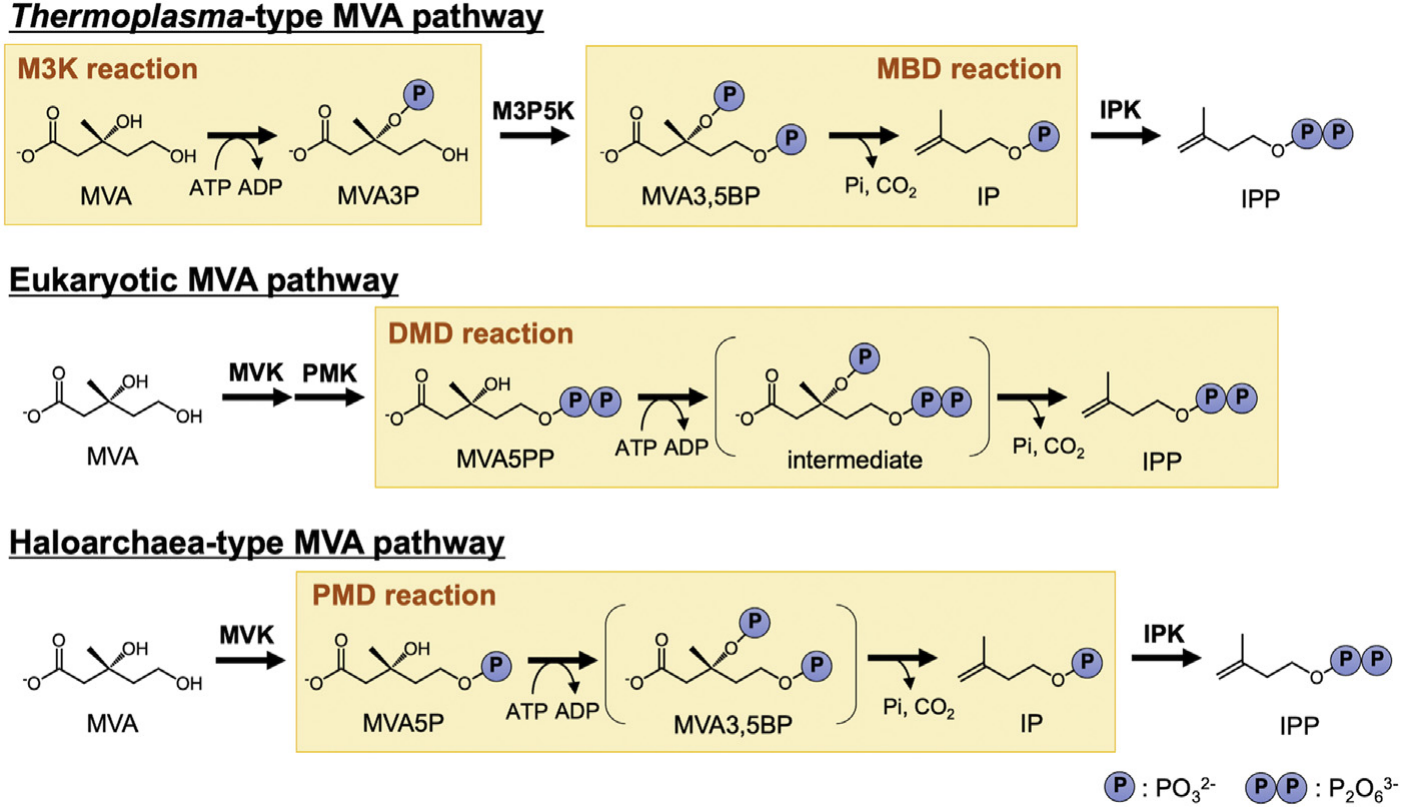
**DMD homolog enzymes involved in the known MVA pathways.** Enzyme reactions of a part of the eukaryotic MVA pathway, the *Thermoplasma*-type MVA pathway, and the haloarchaea-type MVA pathway, from MVA to IPP are indicated. Upstream reactions from acetyl-CoA to MVA and the final isomerase reaction from IPP to DMAPP are omitted for sake of simplicity. The reactions catalyzed by the DMD homologs are boxed. The archeal MVA pathway, which does not involve DMD homologs, was omitted. DMAPP, dimethylallyl diphosphate; DMD, diphosphomevalonate decarboxylase; IPP, isopentenyl diphosphate; MVA, mevalonate.

Interestingly from an evolutionary perspective, the two pathways use a different process for conducting the decarboxylation step *via* homologous enzymes (Fig. 1). In the *Thermoplasma*-type MVA pathway, M3K and MBD work sequentially to carry out the decarboxylation (6). In the eukaryotic MVA pathway, however, one enzyme, DMD, catalyzes dehydration/decarboxylation of MVA 5-diphosphate (MVA5PP) in an ATP-dependent manner to yield IPP (12). M3K, MBD, and DMD share high sequence similarity (1, 6, 7). The DMD reaction is thought to happen in two steps without the release of an intermediate metabolite. First, the enzyme phosphorylates the 3-hydroxyl group of MVA5PP forming a transient intermediate, MVA 3-phosphate 5-diphosphate. Then DMD catalyzes the elimination of the 3-phosphate group of the intermediate, which leads to spontaneous decarboxylation. Thus, in the *Thermoplasma*-type MVA pathway, ATP-dependent phosphorylation of MVA catalyzed by M3K and decarboxylation of MVA3,5BP catalyzed by MBD resemble the entire reaction of DMD but are carried out in two separate steps by two separate enzymes. This splitting of the single enzyme reaction into two enzymes is found only in the *Thermoplasma*-type MVA pathway, which distinguishes the pathway from not only the eukaryotic MVA pathway but also other modified MVA pathways, that is, the haloarchaea-type MVA pathway (2, 5) and the recently discovered archeal MVA pathway (4). In the haloarchaea-type MVA pathway, which is found in halophilic archaea (5) and some Chloroflexi bacteria (2), a DMD homolog, phosphomevalonate decarboxylase (PMD), catalyzes both the ATP-dependent 3-phosphorylation and decarboxylation of MVA 5-phosphate (MVA5P) to produce IP. In contrast, the archeal MVA pathway, which is thought to be used in the majority of archaea, does not involve DMD homologs. The pathway resembles the haloarchaea-type pathway, but the role of PMD to convert MVA5P into IP is carried out by two enzymes, phosphomevalonate dehydratase and *trans*-anhydromevalonate phosphate decarboxylase, which have no sequence similarity with DMD homologs (4). Therefore, the archeal pathway passes through an intermediate, *trans*-anhydromevalonate 5-phosphate, which is absent in the other MVA pathways.

To better understand how M3K and MBD evolved to split the kinase and phosphate elimination/decarboxylation functions that are normally catalyzed by single DMD/PMD homologs, we sought to determine the crystal structure of an MBD enzyme, which is the only missing structure in the set of homologs. The crystal structures of DMDs from several organisms have been solved (12, 13, 14, 15, 16, 17, 18, 19, 20, 21), while a PMD structure has been determined from a Chloroflexi bacterium, *Anaerolinea thermophila* (20). We recently solved the crystal structures of M3K from *Thermoplasma acidophilum* (22). The lack of decarboxylase activity in M3K was explained by the absence of the catalytic aspartate residue in the active site that is conserved among DMDs. The replacement of the aspartate residue was shown to change archeal DMD into a kinase, which only catalyzed 3-phosphorylation and lost phosphate-elimination/decarboxylation activity (12). In contrast, there is no structural information obtained for MBD. Given the high similarity between the amino acid sequences of MBD and DMD, understanding the structural difference that makes MBD not require ATP is of great interest.

In this study, we solved the crystal structure of MBD from a Thermoplasmatales archaeon *Picrophilus torridus* and performed mutagenesis on the enzyme to evaluate the catalytic importance of some amino acids conserved among DMDs/PMDs and likely in *P. torridus* MBD (PtoMBD). Interestingly, the structure revealed a potential lipoidal ligand in a cavity corresponding to the ATP-binding site of DMD. In a search for the physiological ligand for the archeal enzyme, we find that MBD is activated by geranylgeranyl diphosphate (GGPP), a major intermediate of archeal isoprenoid biosynthesis. The data reported here help us understand how these enzymes have evolved and added regulatory functionality.

## Results

### Overall structure of PtoMBD

**Figure 2:**
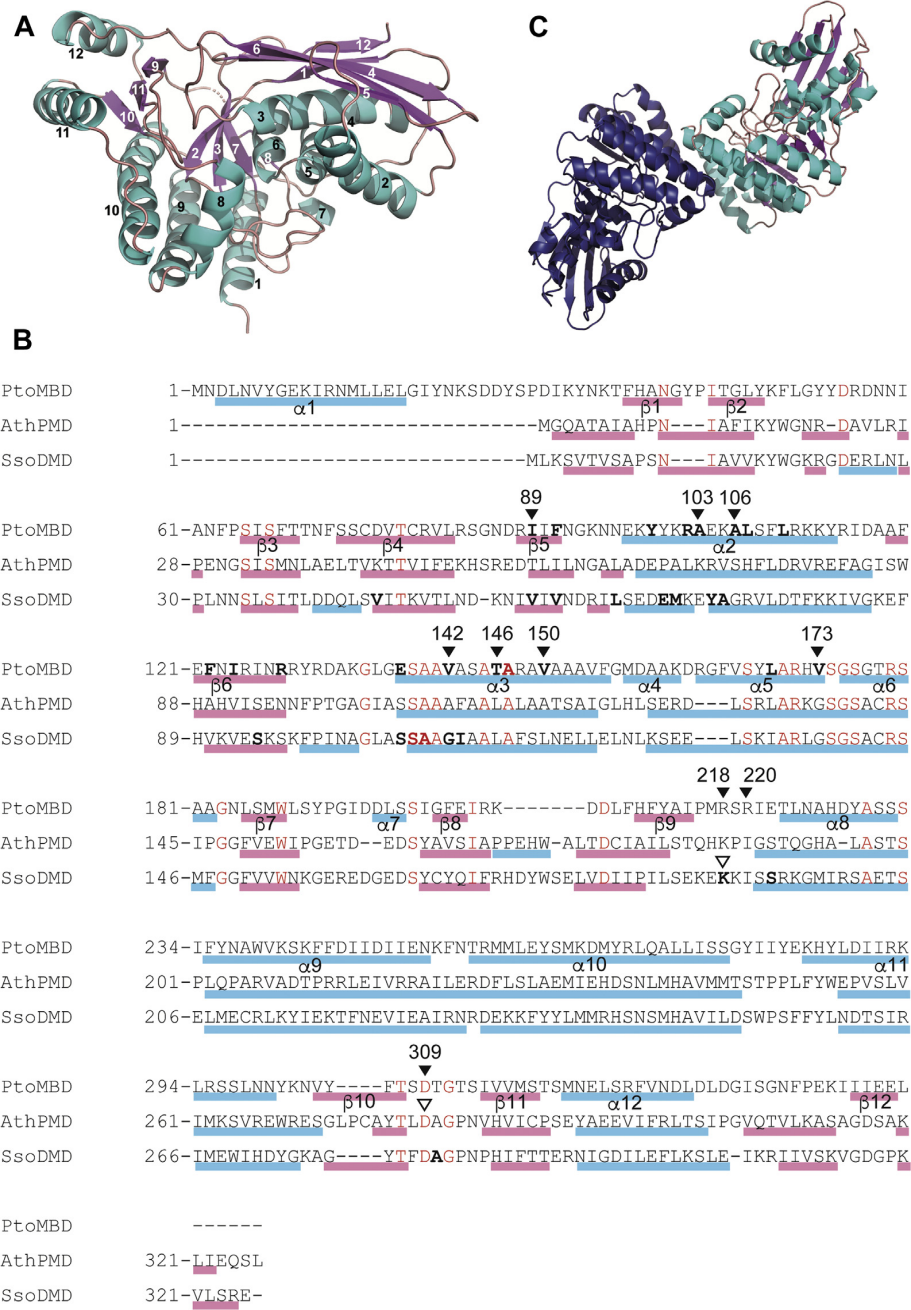
**Structure of PtoMBD.***A*, overall structure. *Black* and *white* numbers represent the numbering of α-helices and β-strands, respectively. *B*, amino acid alignment of PtMBD, *A. thermophila* PMD (AthPMD), and SsoDMD. α-helices and β-strands are indicated by *cyan* and *magenta bars*, respectively. *Bold letters* indicate the amino acid residues that are in close contact (within 5Å) with the ligands, OLA in PtoMBD and ATPγS in SsoDMD. *Closed arrowheads* indicate the residues of PtoMBD that are mutagenized in this study. *Open arrowheads* indicate the residues of AthPMD and SsoDMD corresponding to some of the mutagenized residues, which are shown in Figure 5. *C*, the most likely homodimeric structure of PtoMBD. ATPγS, adenosine 5ʹ-*O*-(3-thio)triphosphate; PtoMBD, *Picrophilus torridus* mevalonate 3,5-bisphosphate decarboxylase; SsoDMD, *Saccharolobus solfataricus* diphosphomevalonate decarboxylase.

The X-ray crystal structure of PtoMBD was solved at 2.2 Å resolution. Two protein molecules are observed in the asymmetric unit of space group P2_1_2_1_2. The two protein chains in the asymmetric unit are essentially identical, with an RMSD of 0.59 Å, excluding disordered regions (residue 206 in subunit A; residues 221 and 222 in subunit B) and the binding of a fatty acid–like ligand described later. The PtoMBD monomers comprise 12 α-helices and 12 β-strands (Fig. 2*A*). The α1 helix, which does not exist in known structures of DMD and PMD, is formed at the extended N-terminal region of PtoMBD (Fig. 2*B*). An antiparallel β-sheet composed of β6-β4-β1-β12 strands forms part of the outer surface of the enzyme with the β5 strand, while β2-β3-β7-β8 strands and β9-β11-β10 strands compose antiparallel β-sheets, which are surrounded by α-helices. The β2-β3-β7-β8-β9-β10-β11 sheets and α1, α8, α9, α10, α11, and α12 helices form the bottom floor of the enzyme, and a large cleft, which corresponds to the substrate-binding sites of the known structures of DMD homologs, exists between the floor and an upper domain composed of α2, α3, α4, α5, α6, and α7 helices and the β5-β6-β4-β1-β12 sheet. This structure resembles those of homologous decarboxylases classified in the GHMP kinase (galactokinase, homoserine kinase, MVK, and PMK) superfamily, that is, DMD and PMD, and also other GHMP superfamily kinases including M3K (6).

The calculation of possible subunit interaction by the PISA program suggested that the most likely biological unit of PtoMBD is a homodimer made between subunit A and B of different asymmetric units of the crystal, not the two chains within an asymmetric unit. The putative dimer interface has a large contact surface area of 1139 Å^2^, made between subunits at helices α9, α10, and α11 (Fig. 2*C*). The identified homodimeric structure and its dimer interface are similar to those proposed for some homologous enzymes such as DMDs from *Staphylococcus epidermidis* (14), *Saccharolobus solfataricus* (19), and *Arabidopsis thaliana*, and PMD from *A. thermophila* (20). Size-exclusion chromatography analysis of PtoMBD with an N-terminal polyhistidine tag revealed a primary peak with retention time corresponding to 75.7 kDa, which is almost consistent with a homodimer of tagged PtoMBD molecules with calculated mass 42,186 Da (Fig. S1).

### Binding of an unknown ligand

**Figure 3:**
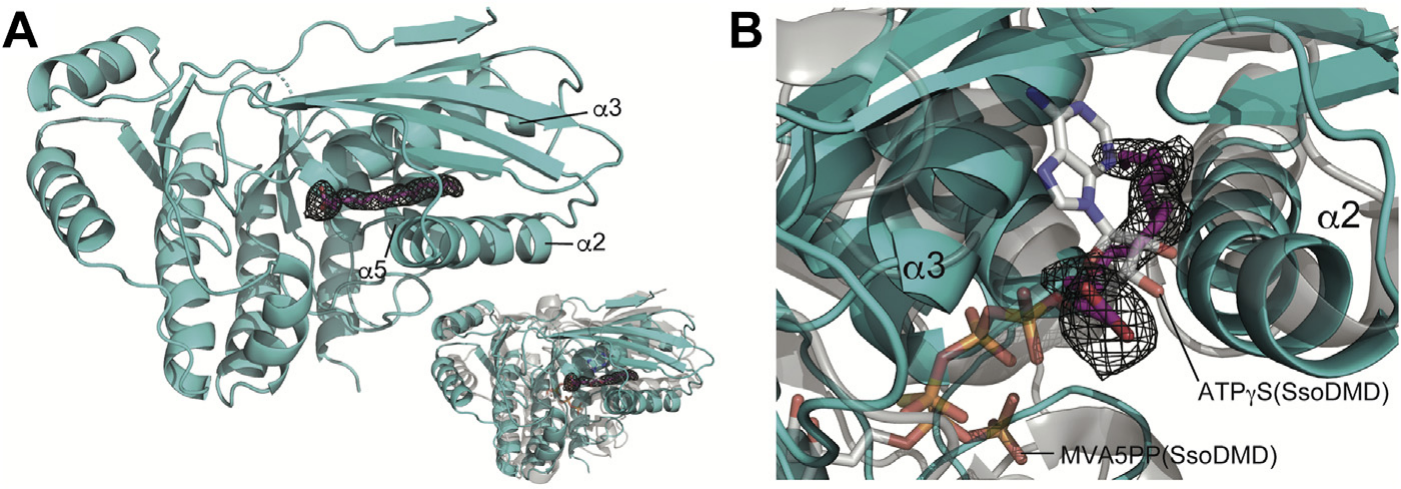
**Ligand binding in PtoMBD.***A*, electron density of the fatty acid–like ligand. The polder map contoured at 5.5 σ in subunit A of the PtoMBD structure is shown in *black*. OLA, which is fitted to the electron density, is shown by stick models in *magenta*. Inset: Superposed monomeric structures of PtoMBD (*cyan*) and SsoDMD (pdb: 5gmd, *gray*). *B*, closeup view of the superposed ligand-binding sites of PtoMBD (*cyan*) and SsoDMD (*gray*). OLA, oleic acid; PtoMBD, *Picrophilus torridus* mevalonate 3,5-bisphosphate decarboxylase; SsoDMD, *Saccharolobus solfataricus* diphosphomevalonate decarboxylase.

In the large cleft of subunit A, a continuous, linear tube of electron density of ∼20 Å length was observed, although with low occupancy (Fig. 3*A*). The electron density extends deeply into a narrow cavity that is surrounded by α2, α3, and α5 helices and the β5-β6-β4-β1-β12 sheet. The internal surface of the cavity is mostly composed of hydrophobic amino acid residues, and the opening of the cavity is surrounded by charged or polar residues including Lys94, Tyr99, Arg128, and Glu138. These observations suggested that the electron density seen in the cavity was an amphipathic molecule such as fatty acid or detergent. However, no detergents were used in the purification and crystallization processes, and no other molecule in the crystallization conditions would fit the shape of this density tube, so we concluded that the unknown ligand in the cavity was likely an endogenous fatty acid from *Escherichia coli*, which was used as the host organism for recombinant protein expression. Oleic acid (OLA) fitted well in the electron density and therefore was included in the structural model of PtoMBD. We also examined the fitting of isoprenoid precursors into the electron density of the ligand, such as farnesyl diphosphate (FPP) and GGPP, but their lengths and shapes, especially at the diphosphate group, did not fit well into the electron density.

Interestingly, the binding site of the fatty acid–like ligand overlaps with the ATP-binding site of DMD, which has been elucidated by the structural studies of DMDs from *S. epidermidis*, *S. solfataricus*, and *Enterococcus faecalis* (12, 13, 17). As shown in Figure 3*B* (and also in Fig. 2*B*), an ATP analog molecule, adenosine 5ʹ-*O*-(3-thio)triphosphate (ATPγS), binds to a cleft surrounded by α2 and α3 helices and β5 and β6 strands (in the numbering of PtMBD) in a structure of *S. solfataricus* DMD (SsoDMD). When the structure of PtoMBD is superposed with that of SsoDMD, the OLA molecule in PtoMBD overlaps the ribose moiety of ATPγS in SsoDMD. This structural information suggests that PtoMBD lacks an ATP-binding site, at least when it binds the fatty acid–like ligand. This finding is compatible with the fact that PtoMBD does not require ATP for reaction.

### Discovery that an isoprenoid ligand activates PtoMBD

**Figure 4:**
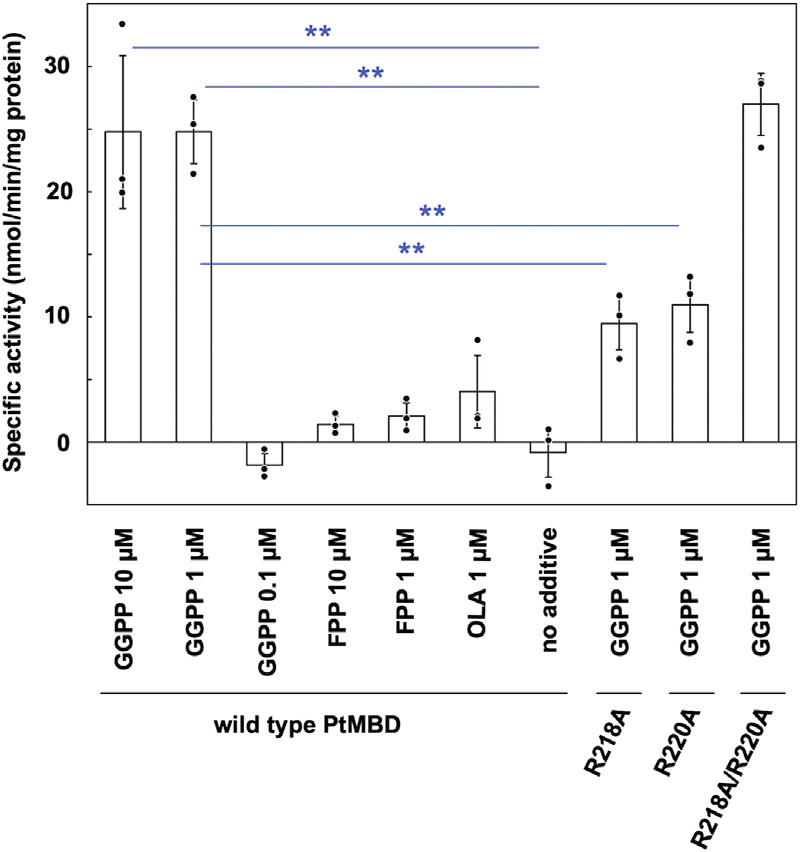
**Effects of the addition of ligands and mutations on the activity of PtoMBD.** The standard assay conditions described in the Experimental procedure section were used for reaction, excepting the addition of ligands shown in the figure. The background activity obtained from the reaction without the enzyme has been subtracted from the data. Open bars and error bars indicate average and SD values of the data (n = 3), respectively. *Double asterisks* above lines between two bars mean significant difference (*p* < 0.01 using Student’s *t* test). The raw data from the TLC analysis was shown in Figure S4. PtoMBD, *Picrophilus torridus* mevalonate 3,5-bisphosphate decarboxylase.

Although we suspect that a fatty acid from the expression host bacterium *E. coli* binds in the cavity of, at least a part of, recombinant PtoMBD, we speculated that a different hydrophobic molecule would be the physiological ligand of PtoMBD because archaea do not usually synthesize fatty acids. We first tested GGPP, which is an amphipathic linear molecule and is considered to be a major biosynthetic intermediate for archeal isoprenoids such as respiratory quinones, dolichols, and archeal membrane lipids (23). As shown in Figure 4, 10 or 1 μM GGPP significantly activated PtoMBD compared to the conditions with no additives, while enzyme activity in the presence of 0.1 μM GGPP was at the background level, below the detection limit of the assay method. When 1 or 10 μM FPP, a shorter isoprenoid precursor, or 1 μM OLA, which is comparable in molecular size to GGPP, was added instead, PtoMBD seemed slightly activated, but the activation was statically insignificant. These data suggest that GGPP or related compounds might bind to MBD as a physiological ligand in the cells of *P. torridus* to activate the enzyme. Thus, we added 1 μM GGPP for all subsequent MBD reactions. The potential physiological meaning of the activation by GGPP, which is a downstream metabolite of the MVA pathway that involves MBD, will be discussed later.

### Mutagenic studies on PtoMBD

**Figure 5:**
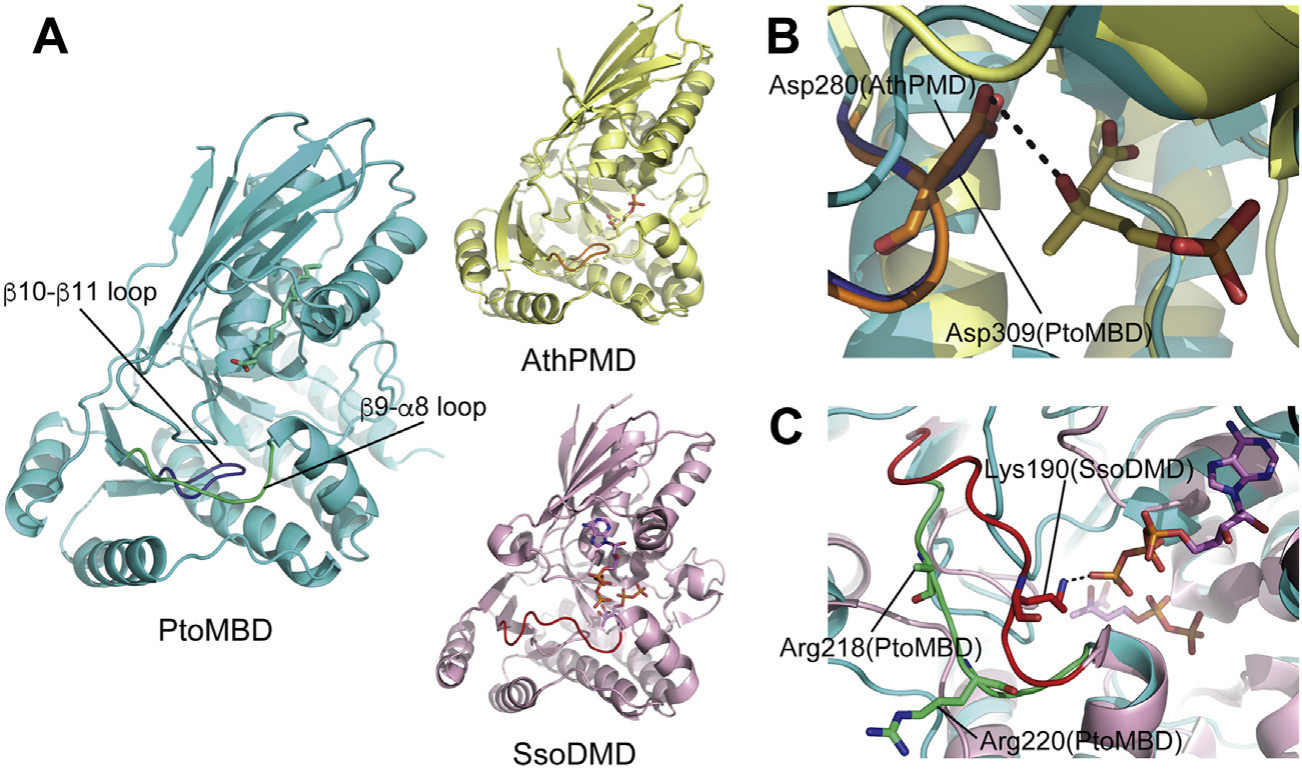
**Mutated amino acid residues that correspond with those required for catalysis by DMD homologs.***A*, comparison of the monomeric structures of PtoMBD (PDB: 7T71, *cyan*), AthPMD binding MVA5P (6n0x, *yellow*), and SsoDMD binding MVA5PP and ATPγS (5gmd, *pink*). The β10-β11 loop of PtoMBD and the corresponding loop of AthPMD are colored in *dark blue* and *orange*, respectively. The β9-α8 loop of PtoMBD and the corresponding loop of SsoDMD are colored in *green* and *red*, respectively. *B*, superposed active-site structures of PtoMBD and AthPMD. The Asp309 residue of PtoMBD, which is mutated in this study, and the corresponding Asp280 residue interacting with the substrate MVA5P in AthPMD are shown in *stick models*. *C*, superposed loop structures of PtoMBD and SsoDMD. Arg218 and Arg220 residues exist on the β9-α8 loop of PtoMBD, while the side chain of Arg218 is disordered. The Lys190 residue of SsoDMD exists on the corresponding loop, which moves largely in response to the binding of ATPγS, and interacts with the γ-thiophosphate group of the ATP analog. AthPMD, Anaerolinea thermophila phosphomevalonate decarboxylase; ATPγS, adenosine 5ʹ-*O*-(3-thio)triphosphate; DMD, diphosphomevalonate decarboxylase;MVA, mevalonate; PtoMBD, *Picrophilus torridus* mevalonate 3,5-bisphosphate decarboxylase; SsoDMD, *Saccharolobus solfataricus* diphosphomevalonate decarboxylase.

Several mutants of PtoMBD were constructed to understand the reaction mechanism of the enzyme. The first set of mutants, D309T, D309V, and D309N, was designed based on our recent study of the reaction mechanism of SsoDMD (12). According to that study, replacement of the catalytic residue Asp281 with threonine or valine caused the loss of decarboxylase function and changed SsoDMD into MVA5PP 3-kinase, while the D281N mutant retained DMD activity. This catalytic Asp residue is conserved in all related decarboxylases such as DMD, PMD, and MBD (Fig. 2*B*) but not in the nondecarboxylating homolog, M3K. Thus, the corresponding Asp309 residue of PtoMBD existing in the β10-β11 loop (Fig. 5, *A* and *B*) was also expected to play a critical role because the enzyme does not have kinase activity and only catalyzes phosphate elimination/decarboxylation. Changing this Asp309 residue through the mutants D309T and D309N leads to the complete loss of MBD activity (Fig. S2), affirming the catalytic importance of the aspartate residue. The aspartate residue might also be important for protein folding or stability because the D309V mutant was not expressed in *E. coli* cells (Fig. S3). The expression level of the D309N mutant was low and that of the D309T mutant was weaker than the WT enzyme.

Another set of mutants, R218A, R220A, and R218A/R220A, was designed to study the roles of basic residues on a loop between β9 and α8 of PtoMBD (Figs. 2*B* and 5, *A* and *C*). According to the crystal structure of SsoDMD in complex with MVA5PP and ATPγS (Protein Data Bank [PDB] #: 5gmd), Lys190 on the corresponding loop probably interacts with the γ-thiophosphate group of the ATP analog and is therefore suggested to guide the γ-phosphate group of ATP toward the 3-hydroxyl group of the substrate MVA5PP to stimulate the phosphotransfer reaction (12). Similar interaction between a Lys residue and ATPγS was reported in the enzyme–MVA5PP–ATPγS ternary complex structure of the D283A mutant of *S. epidermidis* DMD (4dpw) (13). Qiu *et al*. reported that the replacement of the corresponding Lys185 residue of rat DMD with alanine resulted in the loss of enzyme activity (24), which suggests the importance of the basic residue in the enzyme reaction. This idea was also supported by the fact that the alanine replacement of the Arg185 residue of *T. acidophilum* M3K, which exists on the corresponding loop and thus is thought to play a similar function, also caused almost complete inactivation (22). Although PtoMBD has no kinase activity and does not require ATP, it still has two basic residues, Arg218 and Arg220, on the loop, suggesting that the basic residue on the loop might be conserved also in MBD to play roles other than the aid of phosphotransfer. To test this hypothesis, each or both of the arginine residues were replaced with alanine. As shown in Figure 4, however, all the Arg mutants retained significant MBD activity. The activities of the R218A and R220A mutants significantly decreased comparing with that of the WT enzyme, but the R218A/R220A double mutant was as active as the WT, demonstrating that these arginine residues are not required for the reaction of PtoMBD, which no longer involves ATP as a substrate.

Taken together, the results from the two lines of mutagenic studies support the existing understanding of the reaction mechanism of the ATP-dependent decarboxylases such as DMD and PMD. The amino acid residue supposedly involved in phosphotransfer from ATP in DMD/PMD is no longer conserved in MBD, while the residue that probably is involved in phosphate-elimination/decarboxylation in DMD/PMD is also strictly required for MBD.

**Figure 6:**
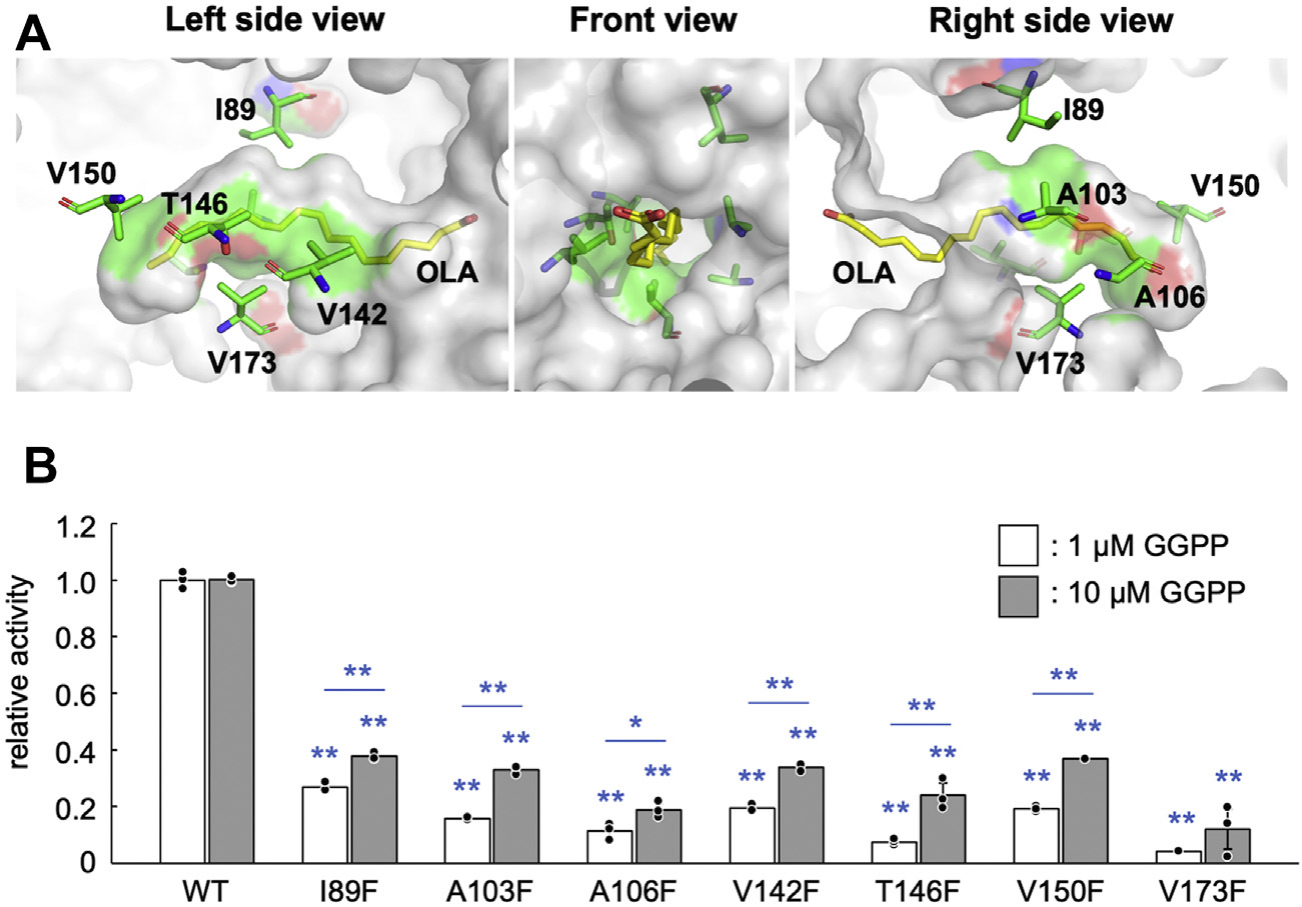
**Phenylalanine replacement mutagenesis of amino acid residues on the surface of the ligand-binding cavity.***A*, the mutagenized amino acids that constitute the wall of the ligand-binding cavity are shown as *green stick* models, and OLA built as a putative ligand is shown as *yellow sticks*. Front view means the view from the opening of the cavity. *B*, activities of PtoMBD mutants, I89F, A103F, A106F, V142F, T146F, V150F, and V173F, in the presence of 1 μM (*open bars*) and 10 μM (*closed gray bars*) GGPP are shown relative to that of the WT enzyme as 1.0. The bars and error bars indicate average and SD values of the data (n = 3), respectively. *Double asterisks* above each bar mean significant difference (*p* < 0.01 using Student’s *t* test) from the activity of the WT enzyme. *Single* and *double asterisks* above lines mean significant difference (*p* < 0.05 and 0.01, respectively) between the relative activities of each mutant in the presence of 1 and 10 μM GGPP. The background activity obtained from the reaction without the enzyme has been subtracted from the data. The raw data from the TLC analysis was shown in Figure S5. GGPP, geranylgeranyl diphosphate;OLA, oleic acid; PtoMBD, PtoMBD, *Picrophilus torridus* mevalonate 3,5-bisphosphate decarboxylase.

A third set of mutants, I89F, A103F, A106F, V142F, T146F, V150F, and V173F, was constructed to determine if GGPP binds to the cavity where the fatty acid–like ligand was observed in the crystal structure of PtoMBD. Several amino acids on the surface of the cavity were selected for mutagenesis (Fig. 6*A*). Changing those amino acids to phenylalanine was intended to narrow different parts of the cavity and hamper the binding of GGPP. The mutants were efficiently expressed in *E. coli* and could be purified by heat treatment and affinity chromatography (Fig. S3). All of them significantly reduced MBD activity compared to the WT PtoMBD, especially in the presence of 1 μM GGPP (Fig. 6*B*), suggesting that GGPP activates MBD by binding to the cavity. Increasing the concentration of GGPP to 10 μM significantly raised the relative activities of some mutants, consistent with presumably higher *K*_D_ values for GGPP in the mutant enzymes.

## Discussion

**Figure 7:**
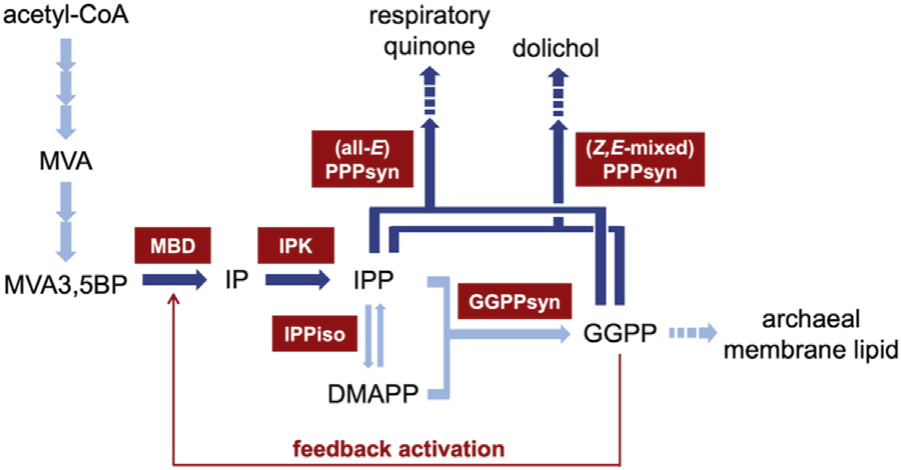
**Feedback regulation system possibly existing in the *Thermoplasma*-type MVA pathway to control downstream isoprenoid metabolism in *P. torridus*.***Dark* and *pale blue arrows* indicate the enzyme reactions whose metabolic flux might be temporarily increased or kept constant through feedback activation of MBD by GGPP, respectively. The names of major enzymes involved are shown in boxes. GGPP, geranylgeranyl diphosphate; GGPPsyn, GGPP synthase; IPPiso, IPP isomerase; MVA, mevalonate; PPPsyn, polyprenyl diphosphate synthase.

The electron density found in the cavity of PtoMBD suggests the binding of a hydrophobic compound, and GGPP at concentration as low as 1 μM was shown to activate the enzyme significantly. Mutagenesis of residues lining the hydrophobic cavity of PtoMBD tended to weaken the activation by GGPP. The role of GGPP binding in the reaction of PtoMBD remains unclear, but the diphosphate moiety of GGPP, which probably exists at the area where the carboxyl group of OLA does, likely intrudes into the active site of the enzyme. The diphosphate group might be directly involved in the MBD reaction as an acid/base catalyst for the elimination of the 3-phosphate group of MVA3,5BP, or its negative charges might stabilize a carbocationic intermediate that is expected to be formed by the 3-phosphate elimination. No matter what role GGPP plays, the findings of the present study suggest that GGPP, which is a major biosynthetic intermediate for archeal isoprenoids, binds to MBD to act as an apparent “feedback activator” of the enzyme. Feedback activation by a downstream metabolite is, however, uncommon in metabolic pathways because it can cause persistent activation and therefore does not contribute to metabolic regulation for homeostasis. One possibility, which would further support these observations, is that the activation of MBD by GGPP controls the stoichiometric balance of the substrates of polyprenyl diphosphate synthases existing downstream of GGPP synthase. The accumulation of GGPP in the cells might indicate a shortage of IPP because (all-*E*) polyprenyl diphosphate synthase, responsible for respiratory quinone biosynthesis and (*Z*,*E*-mixed) polyprenyl diphosphate synthase for dolichol biosynthesis, to utilize both GGPP and IPP as substrates and consume IPP with several times higher rates than GGPP (Fig. 7). In such a situation, a temporary boost of IPP production by the activation of MBD seems reasonable because that would enable the production of respiratory quinone and dolichol, which are required for active growth of the archaeon. Lowering of the GGPP level by the actions of the polyprenyl diphosphate synthases, and also prenyltransferases responsible for archeal membrane lipid biosynthesis, would cancel the activation of MBD and metabolic flow would return to normal. Another possibility is that the binding of GGPP is just a remnant of molecular evolution, as discussed in the next paragraph.

**Figure 8:**
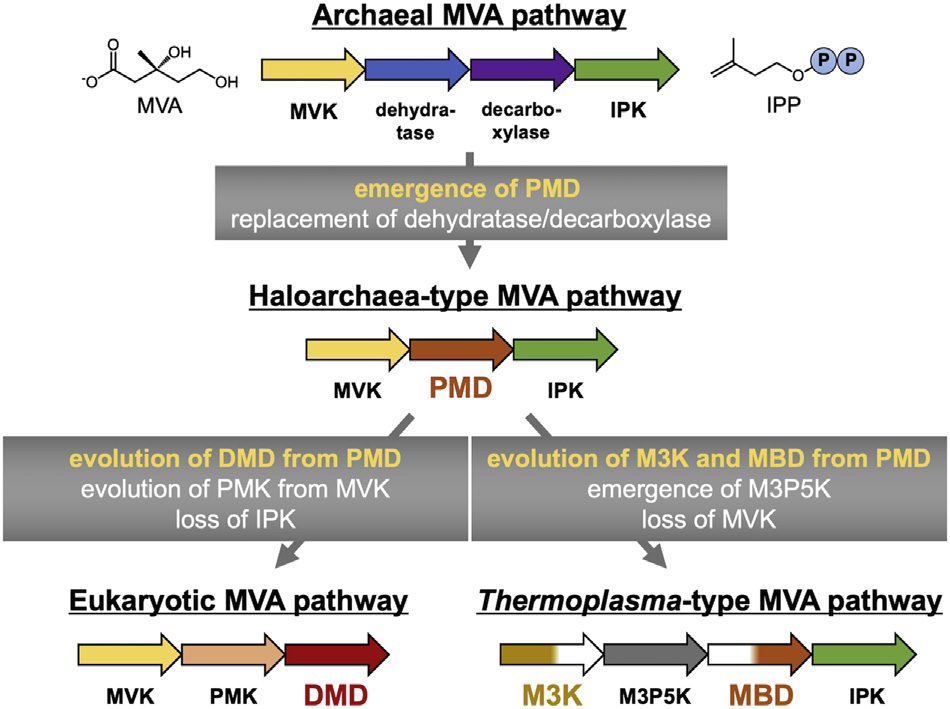
**Hypothetical scheme of the molecular evolution of the MVA pathways.***Arrows* placed from left to right indicate the reactions of enzymes used to convert MVA into IPP in each pathway. The events that are likely to have occurred through the evolution of the pathways are shown in *gray boxes* with slim arrows that indicate the expected direction of evolution. IPP isomerase; MVA, mevalonate.

Molecular evolution of the various MVA pathways, the enzymes involved in the pathways, and regulatory mechanisms working in the enzymes are quite intriguing. If the principle of maximum parsimony is applied, the evolutionary routes of the MVA pathways can be considered as follows (Fig. 8): (1) the haloarchaea-type pathway arose from the prototypic archeal pathway with the emergence of PMD, which is likely the ancestor of all other DMD homologs. PMD replaced the dehydratase/decarboxylase system of the archeal pathway, which also catalyzes the conversion of MVA5P into IP. (2) The eukaryotic pathway evolved from the haloarchaea-type pathway by the molecular evolution of PMD into DMD. Indeed, Thomas *et al*. (20) recently showed that the substrate specificity of PMD can be changed into that of DMD by mutagenesis. In addition, PMK could come into existence *via* the gene duplication of highly homologous MVK. Subsequent loss of the now unnecessary IP kinase would form the well-known, eukaryotic MVA pathway. (3) The *Thermoplasma*-type pathway could diverge from the haloarchaea-type pathway, through the evolution of PMD into M3K/MBD along with the emergence of M3K5P and the loss of MVK. Evolution from PMD to M3K, which would be caused in part by the loss of decarboxylase activity, has been mimicked by our previous mutagenic study that changed archeal DMD into MVA5PP 3-kinase (12). In a similar way, the emergence of MBD from PMD can potentially be experimentally replicated by mutagenesis leading to the loss of kinase activity. Based on the findings that, at low pH, *Roseiflexus castenholzii* PMD cannot catalyze its first half 3-phosphotransfer reaction while retaining the activity of the latter half phosphate elimination/decarboxylation, we have inferred that the evolution of MBD from PMD might occur through adaptation to extremely acidic environments where archaea of genera *Thermoplasma* and *Picrophilus* thrive (6). The activation of MBD by GGPP discovered in the present study suggests that when PMD lost the 3-kinase activity and evolved into MBD, it supposedly recycled the needless ATP-binding pocket for the binding of the downstream intermediate GGPP to compose a feedback regulation system, or it might fill the cavity with the ubiquitous archeal metabolite GGPP just to keep its structure intact.

The regulation of enzyme activity through the binding of prenyl diphosphates such as FPP and GGPP is well known in eukaryotic and bacterial MVKs, while the effect of the ligand is inhibitory (25, 26, 27). Interestingly, the binding position of the ligand in *Streptococcus pneumoniae* MVK, which is a GHMP superfamily kinase related to PtoMBD, overlaps the binding position of ATP (25) and, therefore, agrees well with that of PtoMBD elucidated in this study. The mode of ligand binding can clearly explain the feedback inhibition mechanism of *S. pneumoniae* MVK because the enzyme requires ATP for activity, but it is in sharp contrast with the unclear activation mechanism of ATP-independent PtoMBD. In addition, direct evolutionary relationship between the regulatory systems of the homologous enzymes seems ambiguous because the regulation by prenyl diphosphate is not always conserved in GHMP kinase superfamily enzymes involved in the MVA pathway. Even MVKs from methanogenic archaea were reported to be insensitive to prenyl diphosphates (28, 29), suggesting that the regulatory mechanisms involving the binding of prenyl diphosphates have evolved convergently in PtoMBD and MVKs.

## Experimental procedures

### Materials

(All-*E*) FPP was donated by Dr Chikara Ohto, Toyota Motor Co. All other chemicals were purchased from Sigma–Aldrich, Fujifilm Wako, or Nacalai tesque unless otherwise noted.

### Recombinant expression and purification of PtoMBD

Recombinant expression and purification of PtoMBD were all performed as described previously (6). In summary, *E. coli* BL21(DE3) harboring the pET28a(+) plasmid containing the PtoMBD gene (*pto0478*) was grown in 30 l LB medium supplemented with 50 μg/ml kanamycin at 37 °C overnight, and protein expression was induced with 1.0 mM IPTG. The cells were harvested and disrupted by an Emulsiflex C-3 high pressure homogenizer (Avestin), and the homogenate was centrifuged to recover the supernatant. The resulting supernatant was heat-treated at 60 °C for 2 h, then spun down. The PtoMBD enzyme was purified from the supernatant by nickel-nitrilotriacetic acid (Ni-NTA) affinity chromatography. After elution from the Ni-NTA resin with 250 mM imidazole, the resulting 5 ml of protein solution was passed through a 1 ml HP-Q column to remove negatively charged proteins since PtoMBD is positively charged. The protein solution was then concentrated to 1 ml using a spin filter concentrator with a 10 kDa cutoff. The protein solution was dialyzed to remove imidazole by transferring it into a 1 ml dialysis cartridge with 10 kDa cutoff membrane, which was placed in 2 l of buffer containing 25 mM Bis–Tris, pH 7.0, and 100 mM NaCl. Then the NaCl concentration was increased to 1 M to avoid the precipitation of the purified protein. This solution was used for crystallization. When used for enzyme assay and size-exclusion chromatography analysis, PtoMBD was recombinantly expressed by culturing the same *E. coli* strain in 1 l LB medium supplemented with 50 μg/ml kanamycin at 37 °C for 18 h without IPTG induction and purified only with Ni-NTA affinity chromatography after heat treatment. We did not add IPTG for induction because PtoMBD was expressed without induction probably because of the leaky expression of the T7 polymerase and because IPTG did not increase the recovery of solubilized enzyme. The purity of the enzyme was confirmed by SDS-PAGE with 10% acrylamide gels. The purified PtoMBD fused with an N-terminal polyhistidine-tag was utilized for crystallization.

### X-ray crystallographic analysis

A stock of PtoMBD containing 5.5 mg/ml of the target protein in 25 mM Bis–Tris, pH 7.0, and 1 M NaCl was used to test crystallization conditions *via* the hanging drop method in 96-well plates. For each condition, three 210 nl hanging drops with varying protein stock to reservoir ratios (2:1, 1:1, 1:2) were prepared using a Mosquito nanoliter pipetting robot (SPT LabTech) in the UCLA Macromolecular Crystallization Facility. Large single crystals grew at room temperature after 3 weeks in 2.4 M malonate, pH 7. The crystals were cryoprotected by a quick soak in a solution consisting of 65% reservoir, 35% (v/v) glycerol, then flash frozen in a cryogenic nitrogen stream and maintained at 100 K for data collection.

X-ray diffraction data for the crystals (2.7 Å) were initially collected with a Rigaku FR-E rotating anode X-ray source, using CuKα radiation (λ = 1.5418 Å) and an R-AXIS HTC imaging plate detector. To obtain higher resolution, this crystal was shipped to the Advanced Photon Source (Argonne National Laboratory) for data collection on APS-NECAT beamline 24-ID-C with a DECTRIS PILATUS 6 M detector. Reduction and scaling of data were performed using XDS/XSCALE (30). All datasets were consistent with space group P2_1_2_1_2 (Table 1). The structure of PtoMBD was determined using the automated molecular replacement pipeline, MrBUMP (31). The path that led to a successful solution employed PHASER (32) for molecular replacement using PDB entry 3QT5, *S. epidermidis* DMD, as a search model. The density was refined using Refmac5 (33) with no noncrystallographic symmetry restraints. The residual Fo-Fc map showed clear positive difference density for side chain atoms that were not included in the model, indicating the correctness of the solution. The automatic chain tracing program, Buccaneer (34), built in missing parts of the model. This was followed by building and refinement with the program ARP/wARP (35). After each refinement step, the model was visually inspected in COOT (36) and modified using guidance from both 2Fo-Fc and Fo-Fc difference maps. Data collection and refinement statistics are reported in Table 1. The model was validated with PROCHECK (37), ERRAT (38), and VERIFY3D (39). The coordinates of the final model and structure factors have been deposited in the PDB with PDB code 7T71. All the structural figures in this report were illustrated using the Pymol Molecular Graphics System (version 2.2.0, Schrödinger, LLC). Subunit interaction was calculated by the PISA program provided by PDB in Europe (https://pdbe.org/pisa) (40). The polder omit map in Figure 3 was constructed with the PHENIX-polder map program (41).

**Table 1:** X-ray crystallography statistics (molecular replacement)

	PtoMBD
**PDB Accession Code** **Data Collection**	7T71
Beamline	APS 24-ID-C
Wavelength (λ)	0.9791
Space group	P2_1_2_1_2
Cell dimensions	
*a*, *b*, *c* (Å)	153.6, 125.5, 52.1
α, β, γ (°)	90, 90, 90
Resolution (Å)	2.2 (2.25–2.20)
*R*_*sym*_ (%)	11.1 (87.6)
*I*/σ*I*	15.4 (2.9)
CC (1/2)	99.8 (85.2)
Completeness	99.4 (92.4)
Redundancy	10.8 (9.7)
Wilson B-factor (Å^2^)	41.4
**Refinement**	
Resolution (Å)	2.2
No. reflections	52,436
*R*_work_/*R*_free_	0.184/0.207
**No. atoms**	
Protein	5429
Nonprotein	134
Avg. B-factors (Å^2^)	42.5
**R.M.S.D.**	
Bond lengths (Å)	0.010
Bond angles (°)	1.00
**Ramachandran**	
Favored (%)	97.8
Allowed (%)	1.6
Outliers (%)	0.6

∗One crystal for the structure was used for collection and refinement.

∗Highest-resolution shell is shown in parentheses.

∗*R*_*sym*_ = ∑|I−<I>|/∑<I>, where I is the observed intensity and <I> is the average intensity from observed symmetry-related reflections. CC(1/2) = correlation coefficient between two halves of the data. *R*_work_ = ∑|F_obs_ – F_calc_|/∑F_obs_, where F_obs_ and F_calc_ are the observed and calculated structure factor amplitudes, respectively. *R*_free_ is calculated from 10% of reflections not included in the refinement.

### Cloning, recombinant expression, and purification of *T. acidophilum* M3P5K

The *Ta0762* gene encoding M3P5K was amplified by PCR from the *T. acidophilum* genome using KOD plus DNA polymerase (TOYOBO) using oligonucleotide primers, 5′-cgcgcggcagccatatgatgaactccaggataatgttcatc-3′ and 5′-ggatcctcgagcatatgtcatctcctgtccacaaatttc-3’. The amplified fragment was cloned into NdeI-digested pET15b plasmid using an In-Fusion HD cloning kit (Takara Bio). For the expression of M3P5K, *E. coli* BL21(DE3) harboring the plasmid was cultivated at 37 °C in 1 l LB medium supplemented with 100 μg/ml ampicillin. After 24 h of cultivation without IPTG induction, cells were harvested. Partial purification of the enzymes was performed by affinity chromatography using a 1 ml Histrap FF crude column (GE Healthcare) following the manufacturer’s instruction.

### Preparation of radiolabeled MVA3,5BP

In a 10 μl volume, 4 nmol of [2-^14^C](*R*)-MVA5P (American Radiolabeled Chemicals, Inc, 55 Ci/mol) was hydrolyzed with 7.5 units of Antarctic phosphatase (New England Biolabs) using Antarctic phosphatase buffer supplied by the manufacturer at 37 °C for 24 h. Following heat treatment at 65 °C for 15 min and centrifugation to remove inactivated phosphatase, the supernatant was mixed with 12 nmol of nonlabeled (*R*)-MVA, which was synthesized *via* the hydrolysis of (*R*)-mevalonolactone (Adeka Co) as described elsewhere (1). The solution containing [2-^14^C]MVA of 13.8 Ci/mol was then reacted with 0.73 nmol of M3K from *T. acidophilum* (*Ta1305*), prepared as described elsewhere (1), in a 40 μl volume of reaction mixture containing 2 μmol sodium phosphate buffer, pH7.5, 200 nmol MgCl_2_, and 160 nmol ATP at 60 °C for 3 h. The same reactions were conducted in five tubes, and the reaction mixtures from the tubes were gathered. M3K was removed from the mixture by filtration with a Vivaspin 500 centrifugation filter (10 kDa MWCO, Sartorius). After the first filtration, the filter was washed by additional filtration with 60 μl of water. The filtrates were gathered and then divided into five tubes. The filtrate in each tube was mixed with 0.90 nmol *T acidophilum* M3P5K and 160 nmol ATP, and the 45 μl reaction mixture was incubated at 60 °C for 3 h. The mixtures from the tubes were gathered, and M3P5K was removed from the mixture by filtration with a Vivaspin 500 centrifugation filter. The filtrate was divided into 10 tubes, and 0.2 units of potato apyrase (Nacalai tesque) was added into each tube to hydrolyze ATP and ADP into AMP through incubation at 37 °C for 1 h. After heating at 65 °C for 20 min to inactive apyrase, the reaction mixtures in the tubes were gathered and then centrifuged to remove inactivated apyrase, followed by filtration with a Vivaspin 500 centrifugation filter. The filtrate was linearly spotted on a Silica gel 60 normal-phase TLC plate (GE Healthcare), and the plate was developed with 1-propanol/28% ammonia water/H_2_O (6:3:1). Because the *R*_f_ values of ATP (∼0.05) and ADP (∼0.1) were close to that of MVA3,5BP (∼0.03) under these TLC conditions, the apyrase treatment yielding AMP with *R*_f_ of ∼0.2 was needed to purify MVA3,5BP by TLC. The silica gel at the area of *R*_f_ 0 to 0.1 was scraped from the TLC plate, and radioactivity was eluted from the silica gel with 10 μM sodium acetate. The radioactive solution, which contained 42 nmol of [2-^14^C]MVA3,5BP (13.8 Ci/mol), was used as the substrate for the assay of PtoMBD. At each step of the substrate preparation described previously, the progress of reaction was checked by normal-phase TLC developed with 1-propanol/28% ammonia water/H_2_O (6:3:1). The distribution of radioactivity on the TLC plate was visualized using a Typhoon FLA 9000 imaging analyzer (GE Healthcare).

### Radio-TLC assay of PtoMBD

A typical reaction mixture for the assay of PtoMBD contained, in a 25 μl volume, 0.25 pmol purified PtoMBD, 625 pmol [2-^14^C]MVA3,5BP (13.8 Ci/mol), 1.25 μmol sodium acetate buffer, pH5.5, 125 nmol MgCl_2_, and 25 pmol (all-*E*)-GGPP (Sigma–Aldrich). About 50 μl of mineral oil was added to avoid the evaporation of the reaction mixture. The mixture was incubated at 60 °C for 1 h, and the reaction was stopped by freezing at −80 °C. The overlaid mineral oil was removed while the mixture was frozen. A 10 μl aliquot of the reaction mixture was used for normal-phase TLC analysis performed as described previously. The conversion fraction of the substrate into IP was below 20% in all conditions. The quantification of radioactive spots on TLC plates was performed using the Image Quant TL software (GE Healthcare) attached to the Typhoon FLA 9000 imaging analyzer.

### Mutagenesis of PtoMBD

The plasmids for the expression of the D309T, D309V, D309N, R218A, R220A, I89F, A103F, A106F, V142F, T146F, V150F, and V173F mutants of PtoMBD were constructed by the QuikChange method (Agilent) according to the manufacture’s protocol using the pET28a(+) plasmid containing the PtoMBD gene as a template and the oligonucleotide primers shown in Table 2. The recombinant expression, purification, and the radio-TLC assay of the mutants were performed with the same protocols as described previously for the WT PtoMBD. For the radio-TLC assay of the I89F, A103F, A106F, V142F, T146F, V150F, and V173F mutants, [2-^14^C]MVA3,5BP prepared without apyrase treatment and TLC purification was used as the substrate. Each reaction mixture contained 25 fmol purified PtoMBD and 25 pmol [2-^14^C]MVA3,5BP (55 Ci/mol), while MVA3,5BP contained a significant amount of MVA as a contaminant. Incubation was performed using an Applied Biosystems 2720 Thermal Cycler without the overlay of mineral oil.

**Table 2:** Primers used to construct expression vectors for PtoMBD mutants

Mutants	Primers
D309T	5′-gtgtactttacgagcacaacgggaacgtccatcg-3′ (forward)
	5′-cgatggacgttcccgttgtgctcgtaaagtacac-3′ (reverse)
D309V	5′-gtgtactttacgagcgtaacgggaacgtccatcg-3′ (forward)
	5′-cgatggacgttcccgttacgctcgtaaagtacac-3′ (reverse)
D309N	5′-gtgtactttacgagcaacacgggaacgtccatcg-3′ (forward)
	5′-cgatggacgttcccgtgttgctcgtaaagtacac-3′ (reverse)
R218A	5′-catttttatgcaatccctatggccagccgtatcgaaacac-3′ (forward)
	5′-gtgtttcgatacggctggccatagggattgcataaaaatg-3′ (reverse)
R220A	5′-caatccctatgcgcagcgccatcgaaacacttaac-3′ (forward)
	5′-gttaagtgtttcgatggcgctgcgcatagggattg-3′ (reverse)
I89F	5′-gcgtagcgggaacgaccgctttatttttaacggcaaaaac-3′ (forward)
	5′-gtttttgccgttaaaaataaagcggtcgttcccgctacgc-3′ (reverse)
A103F	5′-caacgaaaaatactataagcgctttgaaaaagctctgtcgttcttgcg-3′ (forward)
	5′-cgcaagaacgacagagctttttcaaagcgcttatagtatttttcgttg-3′ (reverse)
A106F	5′-ctataagcgcgcagaaaaatttctgtcgttcttgcgtaaaaagtacc-3′ (forward)
	5′-ggtactttttacgcaagaacgacagaaatttttctgcgcgcttatag-3′ (reverse)
V142F	5′-gtctcggcgagtccgccgcctttgcatccgctactgcgcgtgc-3′ (forward)
	5′-gcacgcgcagtagcggatgcaaaggcggcggactcgccgagac-3′ (reverse)
T146F	5′-gttgcatccgcttttgcgcgtgcggtc-3′ (forward)
	5′-gaccgcacgcgcaaaagcggatgcaac-3′ (reverse)
V150F	5′-ctactgcgcgtgcgtttgctgccgcggtttttg-3′ (forward)
	5′-caaaaaccgcggcagcaaacgcacgcgcagtag-3′ (reverse)
V173F	5′-cttacctggcgcgccatttttcaggctctgggac-3′ (forward)
	5′-gtcccagagcctgaaaaatggcgcgccaggtaag-3′ (reverse)

### Size-exclusion chromatography

PtoMBD purified with affinity chromatography was buffer exchanged with 20 mM sodium phosphate, pH 7.4, containing 500 mM NaCl and concentrated using a Vivaspin Turbo 4 (10,000 MWCO) ultracentrifugation unit (Merck Millipore). The concentrated enzyme solution was loaded onto a HiLoad 16/600 Superdex 200 prep grade gel-filtration column (GE Healthcare) and eluted with the same buffer at a flow rate of 0.6 ml/min using an ÄKTA pure 25 chromatography system (GE Healthcare). The elution of protein was monitored by UV absorption at 280 nm. A standard curve for calibration was obtained using a gel filtration markers kit (Sigma–Aldrich) containing blue dextran (2000 kDa), thyroglobulin (669 kDa), apoferritin (443 kDa), β-amylase (200 kDa), alcohol dehydrogenase (150 kDa), bovine serum albumin (66 kDa), and carbonic anhydrase (29 kDa) and potassium ferricyanide (Wako).

## Data availability

The structure presented in this article has been deposited in the Protein Data Bank with the following code: 7T71. All remaining data are contained within the article.

## Supporting information

This article contains supporting information.

## Conflict of interest

J. U. B. has founded a company involved in the production of natural chemicals that bears no direct relation to this work. The authors declare that they have no conflicts of interest with the contents of this article.

## Supporting information

supplementary information2.1
