# Supplementary material for: Crystal structure of mevalonate 3,5-bisphosphate decarboxylase reveals insight into the evolution of decarboxylases in the mevalonate metabolic pathways
Source: J Biol Chem. 2022 Jun 9;298(7):102111. doi: 10.1016/j.jbc.2022.102111 (PMC9254496; doi:10.1016/j.jbc.2022.102111)
Supplement: supplementary information2.1 [file mmc1.docx]

**Supplementary information**

Crystal structure of mevalonate 3,5-bisphosphate decarboxylase reveals insight into the evolution of decarboxylases in the mevalonate metabolic pathways

**Authors**

Mizuki Aoki^1,*^, Jeffrey Vinokur^2,*^**,** Kento Motoyama^1^, Rino Ishikawa^1^, Michael Collazo^3^, Duilio Cascio^3^, Michael R. Sawaya^4^, Tomokazu Ito^1^, James U. Bowie^2^, Hisashi Hemmi^1,†^

^1^Department of Applied Biosciences, Graduate School of Bioagricultural Sciences, Nagoya University, Furo-cho, Chikusa-ku, Nagoya, Aichi 464-8601, Japan, ^2^Department of Chemistry and Biochemistry, UCLA-DOE Institute, Molecular Biology Institute, University of California Los Angeles (UCLA), California 90095-1570, USA, ^3^Departments of Biological Chemistry, UCLA-DOE Institute of Genomics and Proteomics, University of California Los Angeles (UCLA), Los Angeles, California 90095, USA., and ^4^UCLA-DOE Institute of Genomics and Proteomics, Howard Hughes Medical Institute, University of California Los Angeles (UCLA), Los Angeles, California 90095, USA.

^*^The first two authors contributed equally and are listed in alphabetical order.

^†^To whom correspondence should be addressed.

Corresponding author

Hisashi Hemmi E-mail: hhemmi@agr.nagoya-u.ac.jp

**
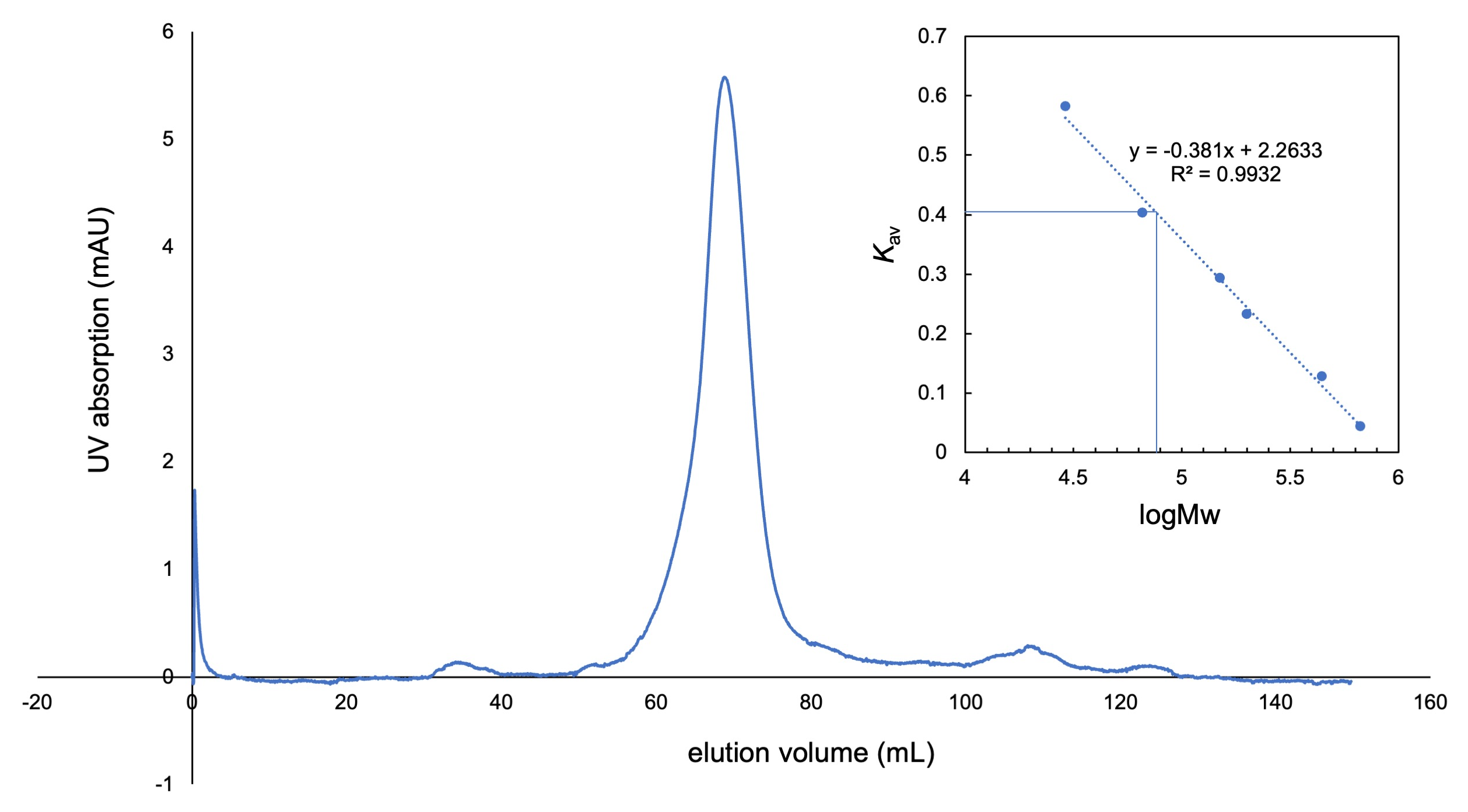
**

**Figure S1:** Size exclusion chromatography analysis of PtoMBD

The elution profile of polyhistidine-tagged PtoMBD from a gel-filtration column was monitored by UV absorption at 280 nm. The standard curve for calibration (inset) was obtained with thyroglobulin (669 kDa), apoferritin (443 kDa), β-amylase (200 kDa), alcohol dehydrogenase (150 kDa), bovine serum albumin (66 kDa), and carbonic anhydrase (29 kDa), using blue dextran (2,000 kDa) and potassium ferricyanide for the calculation of *K*_av_.


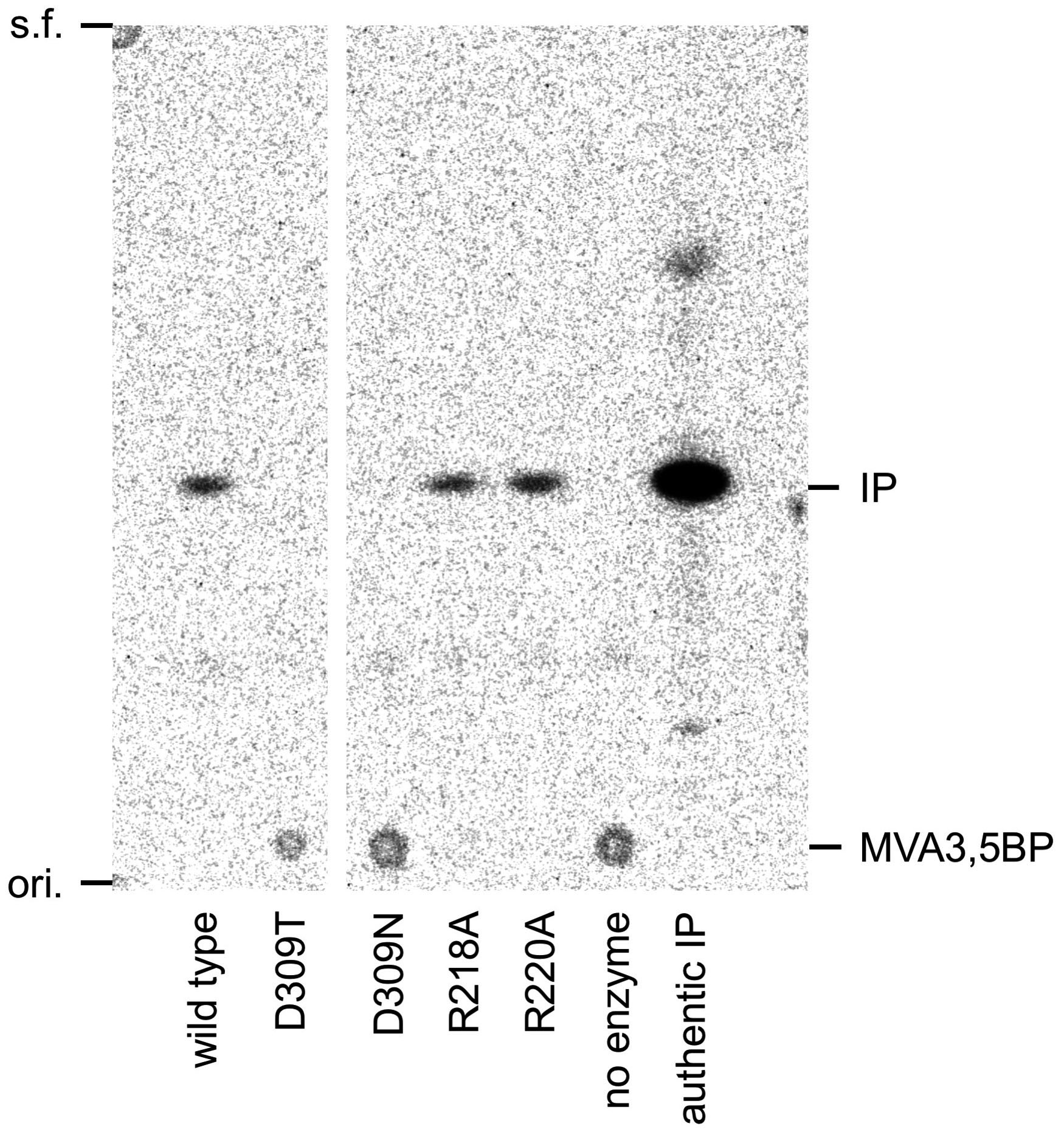


**Figure S2:** TLC analysis of the products from MBD reaction with an excess amount of the wild type or mutant enzymes

Comparable amounts of the enzyme and substrate, 2.5 pmol purified PtoMBD and 3.4 pmol [2-^14^C]MVA3,5BP (55 Ci/mol), were reacted in the presence of 1 µM GGPP. A 25 µL reaction mixture was incubated at 60°C for 1 h, and a 10 µl aliquot of it was used for normal-phase TLC analysis. ori, origin; s.f., solvent front.


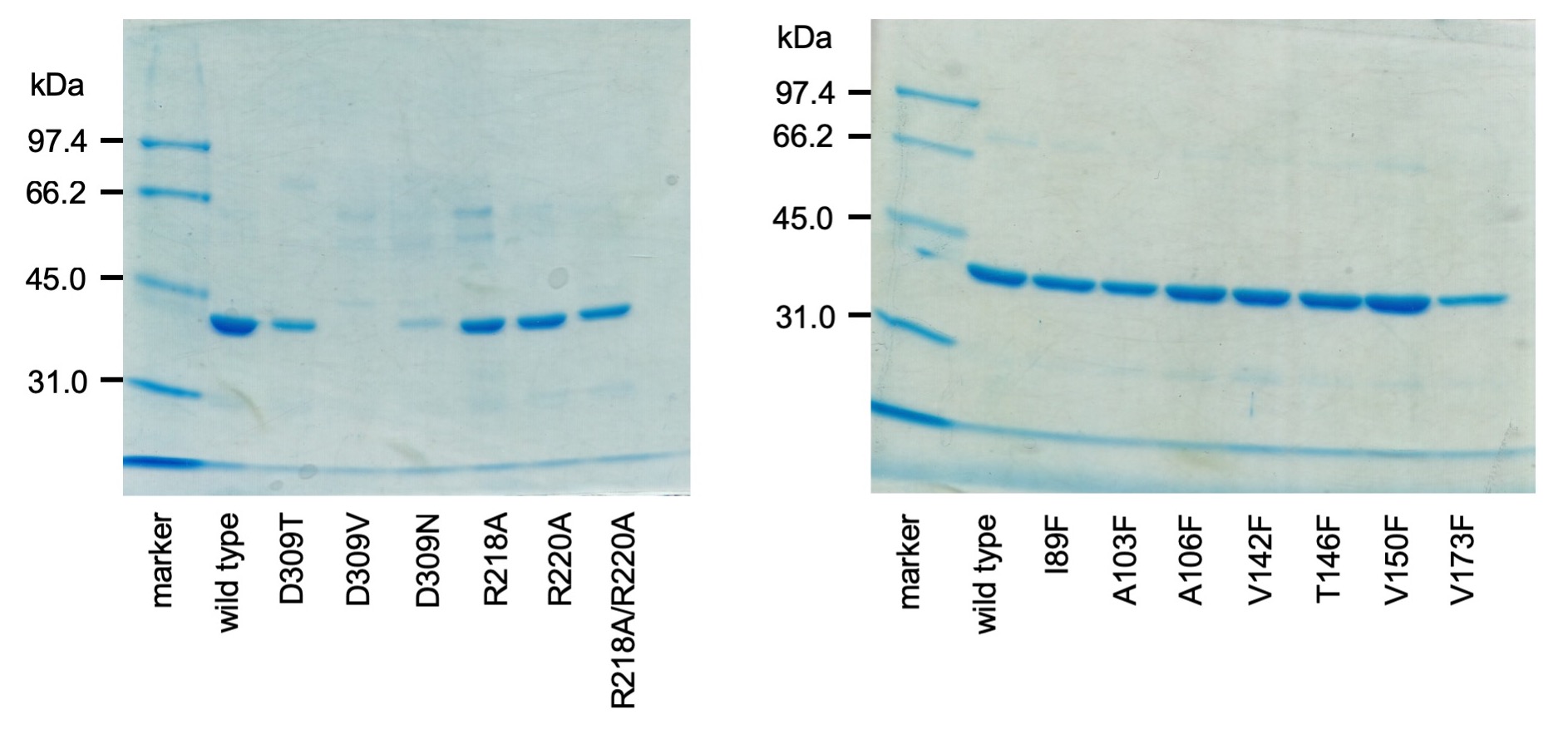


**Figure S3:** SDS-PAGE of the purified mutant PtoMBDs


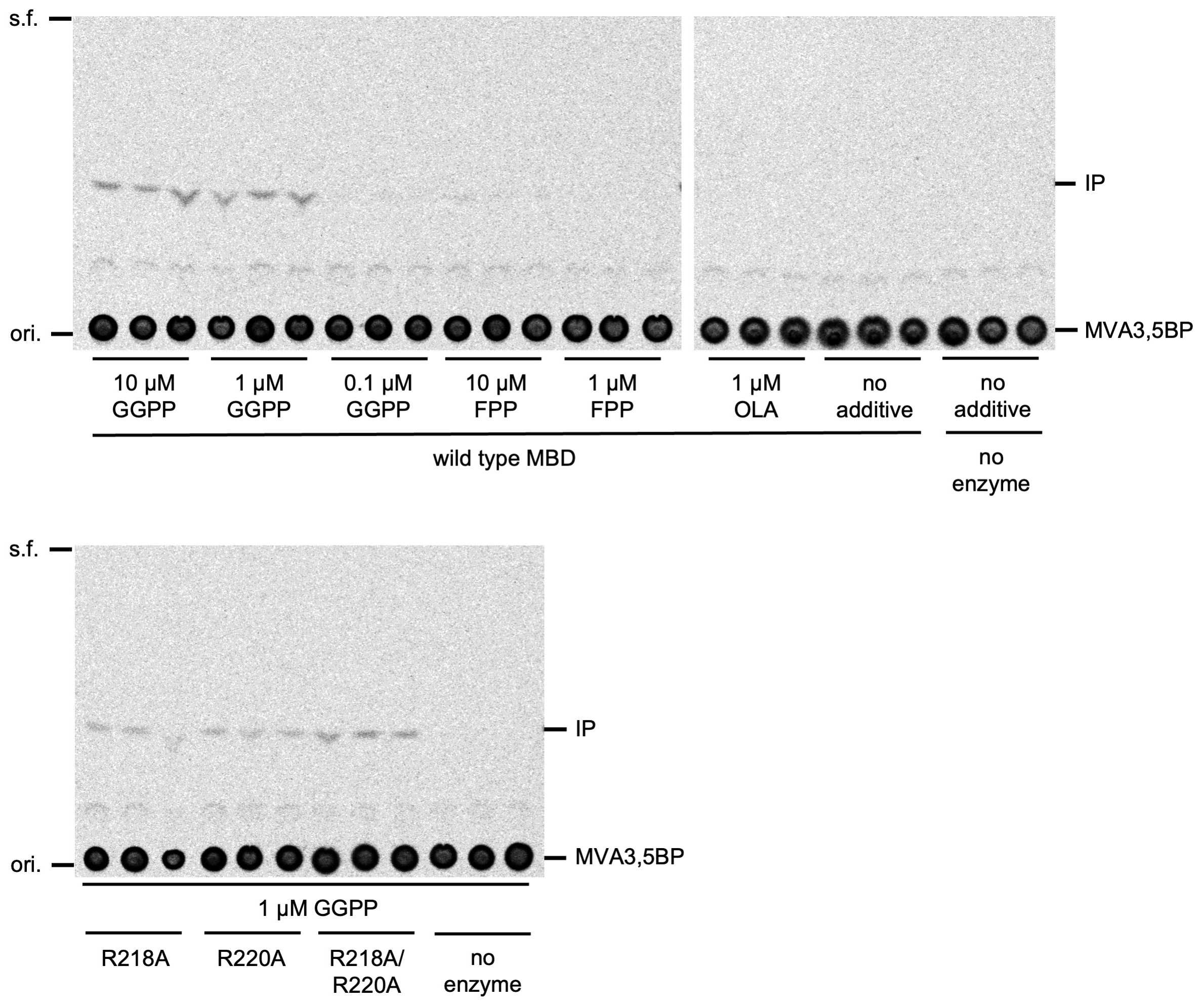


**Figure S4:** Raw data from TLC analysis for Fig. 4

In this assay, 0.25 pmol of purified PtMBD was reacted with 625 pmol [2-^14^C]MVA3,5BP (22 Ci/mol) to obtain the initial rates of reactions.


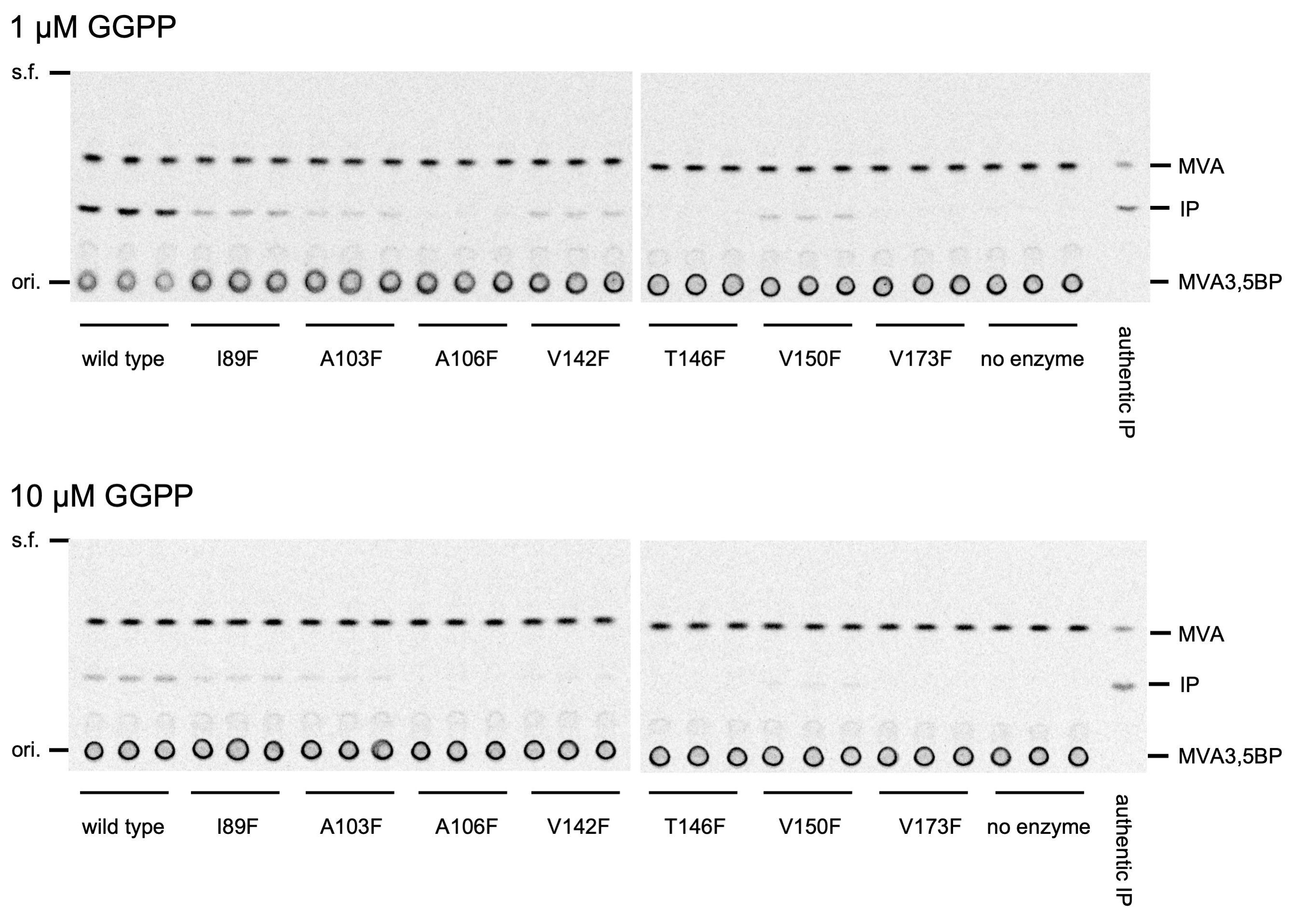
**Figure S5:** Raw data from TLC analysis for Fig. 6B

In this assay, 25 fmol of purified PtMBD was reacted with 25 pmol [2-^14^C]MVA3,5BP (55 Ci/mol) in the presence of 1 (upper panel) or 10 µM GGPP (lower panel). The substrate MVA3,5BP was contaminated by a significant amount of MVA, which was not recognized as the substrate by MBD, because the substrate without apyrase treatment and TLC purification was used for this assay. Authentic IP was formed by the reaction of the substrate with an excess amount of the wild type MBD.
